# Characteristics of adult membranous nephropathy patients with capillary co-deposition of IgG and IgA

**DOI:** 10.1080/0886022X.2025.2512393

**Published:** 2025-06-24

**Authors:** Yi Guan, Aiping Liu, Qionghong Xie, Ruiying Chen, Shaojun Liu, Lingyun Lai

**Affiliations:** a Division of Nephrology, Huashan Hospital, Fudan University, Shanghai, China; b Clinical Laboratory of Huashan Hospital, Fudan University, Shanghai, China

**Keywords:** Membranous nephropathy, IgG, IgA, capillary co-deposition

## Abstract

**Background:**

Membranous nephropathy (MN) is characterized by subepithelial immune complex deposits of IgG, particularly anti-Phospholipase A2 Receptor (anti-Pla2r). However, presence of IgA co-deposition along subepithelial region of the glomerular capillary walls has not been extensively investigated.

**Methods:**

Five hundred thirty-one biopsy-proven MN patients from Huashan Hospital were screened for capillary IgA deposition. Clinical, biological and histopathological data were collected at biopsy and during follow-ups. A propensity score matched cohort was used to compare treatment response and outcomes between MN patients with capillary co-deposition of IgG and IgA (cIgA-MN) and MN without. Immunofluorescence was used to detect IgA, IgG, Galactose deficient-IgA1 (Gd-IgA1) and Pla2r deposition. ELISA was performed to measure circulating Gd-IgA1 level. IgA and IgG subtypes of sera anti-Pla2r antibodies were investigated by indirect immunofluorescence and validated by ELISA.

**Results:**

Fifty-three of 531 MN patients exhibited capillary IgA co-deposition with IgG. Secondary causes were identified in 19 of 53 cIgA-MN patients. The remaining 34 primary cIgA-MN patients had median proteinuria of 6.1 g/day and showed no crescents and mild endocapillary proliferation. 73.5% cIgA-MN patients were positive for Pla2r. Gd-IgA1 was absent in glomeruli of cIgA-MN patients and circulating Gd-IgA1 levels were similar to those in MN patients without IgA deposition. Meanwhile, circulating IgA-type anti-Pla2r antibodies were detected in a subset of cIgA-MN patients. Outcome analysis revealed comparable remission rates and progression to end-stage kideny disease between cIgA-MN and MN without IgA deposition.

**Conclusion:**

This study highlights clinical and pathological features of cIgA-MN, suggesting complex immune responses involving IgA in MN. IgA co-deposition did not significantly alter treatment responses or long-term outcomes.

## Introduction

Among glomerular diseases, the most common types of IgA deposition in the glomerulus are observed in IgA nephropathy (IgAN) and IgA vasculitis nephritis, with deposition primarily occurring in the mesangial area [1]. Other reports of IgA deposition in glomeruli include lupus nephritis, proliferative glomerulonephritis with monoclonal IgA deposits (PGNMID) [2], and IgA-dominant infection-related glomerulonephritis [3], with deposit generally detected in the mesangial and subendothelial regions and in subepithelial regions in some cases. Moreover, IgA deposits have also been detected in the glomeruli of patients with membranous nephropathy (MN).

MN has been recognized as one of the most prevalent etiologies of nephrotic syndrome (NS) in the adult population [4]. It arises from the accumulation of immune complexes within the subepithelial layer of the glomerular basement membrane (GBM) [5] and is characterized by subepithelial immune complex deposits along the glomerular capillary walls, eventually culminating in podocyte injury. Among the various immunological factors implicated in its pathogenesis, anti-Phospholipase A2 receptor (Pla2r) IgG antibodies, particularly those of the IgG4 subclass, have been identified as key components [6]. While IgG antibodies play a vital role in the pathogenesis of primary MN, studies documenting the deposition of IgA antibodies in the subepithelial region of the glomerular capillary walls are scarce.

However, the results of this study indicated that IgA can also be deposited on the capillary wall with IgG. While IgG4 autoantibodies targeting Pla2r remain the hallmark of MN, emerging evidence suggests greater heterogeneity in immune complex composition than previously recognized. Notably, IgA deposition, a feature classically associated with IgA nephropathy, has been anecdotally observed in MN cases but not systematically investigated. Our preliminary observations revealed a distinct subset of MN patients exhibiting co-deposition of IgA with IgG along the glomerular capillary walls, a finding that challenges the conventional IgG-centric paradigm of MN pathogenesis. This novel immunophenotype, which was termed capillary IgA/IgG co-deposition MN (cIgA-MN), raises critical questions. Therefore, this study aimed to characterize the clinicopathological features of adult cIgA-MN patients and elucidate the underlying disease pathogenesis using disease-specific biomarkers. The findings of the present study are expected to raise awareness of this atypical clinicopathological manifestation. Specifically, the unique deposition pattern may reflect exposure to previously unknown epitope spreading in the context of Pla2r-associated MN. Consideration of this phenomenon in this clinical context is crucial, given that different treatment strategies may lead to different prognoses.

## Methods

### Patients

A total of 531 patients with biopsy-proven MN were screened for glomerular IgA staining in the Division of Nephrology at the Huashan Hospital, Fudan University, from January 2007 to May 2020. The intensity of the IgA staining signals was scored as -, ±, +, ++, +++, representing negative, weak, moderate, strong, and very strong signals, respectively. IgA-positive staining was defined as an intensity score of at least +. IgA staining pattern and area were also recorded. Secondary causes of MN, such as malignancy, systemic lupus erythematosus, any type of hepatitis, exposure to toxic agents, and monoclonal gammopathy of undetermined significance or renal significance, were assessed at both admission and last follow-up visit. Pla2r-associated MN was defined as the presence of either Pla2r positivity in renal biopsy or serum specimens. Other participants included those with primary IgAN, MN without capillary IgA co-deposition, and focal segmental glomerular sclerosis (FSGS), with healthy individuals serving as controls. This study was approved by the Ethics Committee of the Huashan Hospital, Fudan University (ethics approval number: KY2016-394) and conducted in accordance with the principles of the Declaration of Helsinki. All patients provided informed consent for the anonymous use of data and samples.

### Clinical and histopathological data

Clinical, biological, and histopathological data at biopsy and the last follow-up visit were reviewed thoroughly. Data were collected according to the standard clinical pathway. The eGFR was calculated using the 2021 CKD-EPI formula [7]. Immunosuppressive therapy included glucocorticoids, calcineurin inhibitors, cyclophosphamide, or rituximab. Complete remission was defined as proteinuria <0.3 g/day, while partial remission was defined as proteinuria <3.5 g/day and a 50% reduction from its peak value [8].

Direct IF for IgA (1:50, F0204, DAKO), IgG (1:50, F0202, DAKO), IgM (1:50, F0203, DAKO), C3 (1:50, F0201, DAKO), C1q (1:50, F0254, DAKO) and IgG1-4 (1:50, Sigma, F0767, F4516, F4641, F9890 respectively) were performed on frozen sections of fresh kidney tissue. Briefly, after incubation for 1 h at room temperature, the slides were washed, mounted, and observed following standard renal biopsy procedures.

### Gd-IgA1 detection

Glomerular Gd-IgA1 deposition was examined by Gd-IgA1 immunofluorescence staining on paraffin-embedded renal biopsy specimens. Briefly, deparaffinized sections were treated with pepsin (Maixin Biotechnologies, Fujian, China) at 37 °C for 40 min for antigen retrieval. Next, the sections were rinsed with phosphate-buffered saline (PBS), followed by incubation with rat monoclonal anti-human Gd-IgA1 antibody (1:50, KM55, anti-human rat IgG monoclonal antibody, #10777, Immuno-Biological Laboratories) at 37 °C for 60 min. Then, after several washes with PBS, the sections were incubated with Cy3-labeled Goat Anti-Rat IgG (Jackson, 1:200) secondary antibody for 1 h at room temperature and imaged under a Biorevo fluorescence microscope (BX53, Olympus Corporation, Tokyo, Japan) equipped with a spot cam digital camera.

Plasma samples were collected on the day of the kidney biopsy, prior to the initiation of immunosuppressive therapy. The samples were stored in aliquots at −80 °C. Circulating Gd-IgA1 levels were detected using a lectin enzyme-linked immunosorbent assay (ELISA) with a commercially available kit (#27600, Immuno-Biological Laboratories) according to the manufacturer’s instructions.

### Pla2r detection

Glomerular Pla2r deposition was examined by immunofluorescence staining for Pla2r on paraffin-embedded renal biopsy specimens. Anti-Pla2r IgG subclasses were detected on frozen sections. For Pla2r staining, the sections were prepared as described above and incubated with Pla2r-Ab (HPA012657, Lot Number 000020610, Sigma, anti-human rabbit IgG polyclonal antibody, 1:500) followed by Cy3-donkey anti-rabbit IgG antibody (Jackson, 1:200). For the detection of anti-Pla2r IgG subclasses, frozen sections was incubated with monoclonal fluorescein isothiocyanate (FITC)-conjugated anti-human IgG1, IgG2, IgG3, and IgG4 antibodies (Sigma, 1:50) at 37 °C for 60 min.

Circulating anti-Pla2r antibodies and their subtypes were detected using indirect immunofluorescence as outlined in a previous study [9] and commercial ELISA kits (EUROIMMUN AG, Lübeck, Germany). Briefly, for the indirect immunofluorescence assay, HEK293 cells transfected with a plasmid containing complementary DNA encoding a full-length Pla2r isoform 1 (origene, accn: NM_007366.3) were incubated with patient serum samples diluted 1:10 in phosphate-buffered saline overnight at 4 °C. FITC-conjugated mouse anti-human IgG and IgA antibodies (Gene Tech) with 1:100 dilution were used for the detection of bound IgG and IgA antibodies, respectively. Transfected cells with specific cytomembrane fluorescence were considered positive. The same assay was performed using serum from FSGS patients and HEK293 cells transfected with a control plasmid, as well as HEK293 cells incubated with commercial anti-Pla2r antibody, which served as negative controls. For ELISA, the presence of anti-Pla2r IgG and IgA antibodies in sera was determined according to the manufacturers’ instructions using enzyme-conjugated secondary antibodies for IgG and IgA, respectively. Anti-Pla2r-IgG positivity was defined as concentrations >20 U/mL, while anti-Pla2r-IgA results were expressed in absolute optical density (O.D.) values. The cutoff O.D. value for anti-Pla2r-IgA was 0.129, corresponding to the mean + 3SD values of healthy controls (*n* = 24).

### Statistical analysis

Data were presented as medians with interquartile ranges for continuous variables or as counts with percentages for categorical variables. Differences between categorical data were analyzed using the Pearson Chi-squared test, Fisher’s exact test, or Mantel-Haenszel χ^2^ test. Differences between quantitative data were analyzed using the Mann-Whitney U test or the Kruskal-Wallis H test. Propensity score matching was performed based on age, gender, and initial proteinuria levels. eGFR was incorporated as a predictor, considering that risk stratification in primary membranous nephropathy predominantly relies on proteinuria, serum albumin, and eGFR, with proteinuria correlating with serum albumin levels. The matching tolerance/caliper was set at 0.03, which was the minimal value necessary to match sufficient patients from a cohort of 180 iMN patients without capillary IgA deposition. Spearman’s correlation analysis was conducted for quantitative and categorical data. Kaplan-Meier plots were generated to illustrate outcomes, and statistical significance was evaluated using log-rank tests. All tests were two-tailed, and *p* < 0.05 was considered statistically significant. Prism version 9.0 (GraphPad Software, La Jolla, CA, USA) and SPSS for Mac version 25 (IBM, Armonk, NY, USA) were employed for statistical analyses.

## Results

### Baseline characteristics of patients

**Figure 1.**
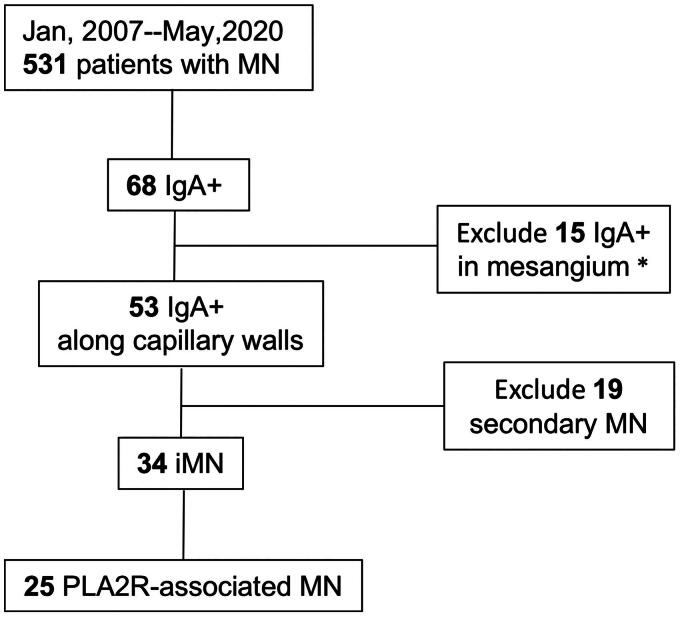
Flowchart of patients selection. Sixty-eight out of 531 biopsy-proven MN patients were screened positive for IgA deposition. Fifteen patients had IgA staining in the mesangial region and excluded. Fifty-three patients had IgA co-deposits along subepithelial region of the glomerular capillary walls with IgG (cIgA-MN). Excluding 19 secondary cIgA-MN patients, 34 primary cIgA-MN were enrolled for further analysis and 25 of them were Pla2r positive. *IgAN combined MN were were diagnosed in 3 patients

In this retrospective study, a total of 531 biopsy-proven MN patients from January 2007 to May 2020 at our department were screened for IgA staining under immunofluorescence microscopy. Mesangial IgA staining was identified in 15 patients, while IgA along the capillary walls with IgG was detected in 53 patients (Figure 1). Secondary causes were identified in 19 of 53 cIgA-MN patients, consisting of ten cases of autoimmune disease, four cases of Hepatitis B infection, three cases of kidney transplantation, one case of chemical exposure, and one case of AL amyloidosis-associated secondary MN (Supplementary Table 1). The remaining 34 idiopathic cIgA-MN patients were included for further analysis, although 3 withdrew from the study during follow-up.

As listed in Table 1, 67.6% of cIgA-MN patients were male, with a median age of 50.5 years (IQR 29.8-64.5). The median proteinuria and eGFR values were 6.1 (IQR 2.2-11.6) g/day and 100.0 (IQR 84.5-119.3) mL/min/1.73 m^2^, respectively. Nephrotic syndrome was present in 67.6% of patients, whilst microscopic hematuria was detected in 94.1% of patients. A total of 25 patients (73.5%) were positive for either renal Pla2r staining or circulating serum anti-Pla2r antibody. Serum IgA, C3, and C-reactive protein levels were within the normal range. Four cIgA-MN patients had a documented history of mild and self-limited upper respiratory tract disease prior to disease onset.

**Table 1.** Clinical characteristics of iMN patients with capillary IgA deposition. *n* = 34.

Baseline characteristics	Patients *N* = 34
Age (years)	50.5 (29.8, 64.5)
Men, n(%)	23 (67.6)
Hypertension, n(%)	10 (29.4)
Diabetes, n(%)	1 (2.9)
Prodromic infection	7 (20.6)
Microscopic hematuria, n(%)	32 (94.1)
Nephrotic syndrome, n(%)	23 (67.6)
Urine erythrocytes (/μL)	61.3 (32.1,102.2)
Initial proteinuria (g/day)	6.1 (2.2, 11.6)
eGFR (mL/min/1.73 m^2^)	100.0 (84.5,119.3)
Pla2R positive, n(%)	25 (73.5)
**Serologic examination**	
Serum albumin (g/L)	26.0 (20.9.33.3)
Serum IgA (g/L)	2.0 (1.6,2.6)
Serum IgG (g/L)	6.1 (4.3,7.8)
Serum IgM (g/L)	0.9 (0.6,1.3)
C3 (g/L)	1.1 (1.0,1.2)
C4 (g/L)	0.24 (0.22,0.30)
C Reaction Protein (mg/L)	3.2 (3.1,3.5)

Data are presented as median (interquartile-range) or number of cases (% of total cases).

eGFR = estimated glomerular filtration rate by EPI equation.

The pathological features are summarized in Table 2. No crescents were observed in cIgA-MN patients. Mild endocapillary proliferation was detected in four (11.8%) patients, and endocapillary proliferation of varying degrees was noted in 27 (79.4%) patients. The number of patients in stages I to IV MN were 11 (32.4%), 14 (41.2%), 8 (23.5%) and 1 (2.9%), respectively. As anticipated, electron microscopy displayed electron-dense deposits in the subepithelial region rather than the mesangial region.

**Table 2.** Pathological characteristics of iMN patients with capillary IgA deposition.

**Histopathological characteristics**	
Percentage of global sclerosis(%)	5.7 (0.0, 13.2)
Percentage of segmental sclerosis (%)	0.0 (0.0,7.1)
Percentage of crescents (%)	0.0 (0.0, 0.0)
Mesangial hypercellularity	
None, n(%)	7/34 (20.6%)
Mild, n(%)	18/34 (52.9%)
Moderate, n(%)	8/34 (23.5%)
Severe, n(%)	1/34 (2.9%)
Endocapillary proliferation	
None, n(%)	30/34 (88.2%)
Mild, n(%)	4/34 (11.8%)
Moderate and severe, n(%)	0/34 (0.0%)
Interstitial fibrosis and tubular atrophy	
None, n(%)	9/3426.5 (%)
Mild, n(%)	17/34 (50.0%)
Moderate, n(%)	5/34 (14.7%)
Severe, n(%)	3/34 (8.8%)
Inflammatory cell infiltration	
None, n(%)	13/34 (38.2%)
Mild, n(%)	12/34 (35.3%)
Moderate, n(%)	6/34 (17.6%)
Severe, n(%)	3/34 (8.8%)
**Glomerular lesion**	
Stage I, n(%)	11/34 (32.4%)
Stage II, n(%)	14/34 (41.2%)
Stage III, n(%)	8/34 (23.5%)
Stage IV, n(%)	1/34 (2.9%)
**Immunofluorescence microscopy rescults**	
IgA, n(%)	34/34 (100%)
IgG, n(%)	34/34 (100%)
IgM, n(%)	11/34 (32.4%)
C3, n(%)	32/34 (94.1%)
C1q, n(%)	10/34 (29.4%)

Data are presented as median (interquartile-range) or number of cases (% of total cases).

### Gd-IgA1 detection

**Figure 2.**
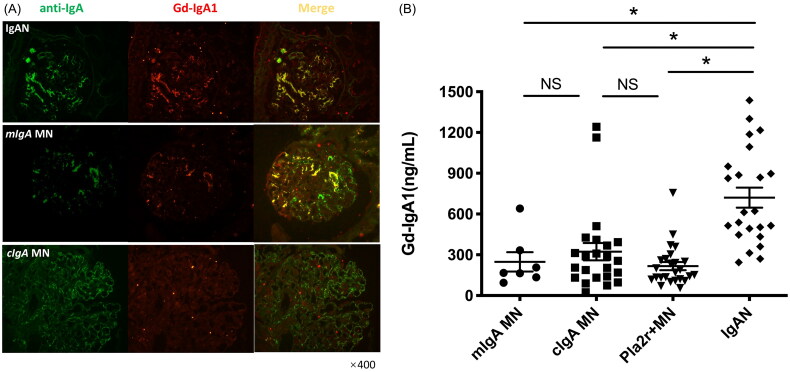
Glomerular staining and plasma levels of Gd-IgA1 in MN patients with mesangial or capillary IgA deposition and primary IgAN patients. (A) Double staining of anti-IgA and KM55 antibody was performed on paraffin sections from renal biopsy of patients diagnosed IgA nephropathy and membranous nephropathy with IgA positive in mesangium (mIgA MN) or along subepithelial region of the glomerular capillary walls (cIgA MN). (B) Plasma KM55 levels in idiopathic MN case with or without IgA deposit and IgAN cases. mIgA MN, idiopathic MN patients with IgA deposit in mesangium, n = 7; cIgA MN, idiopathic MN patients with IgA deposit along capillary walls, n = 23; PLA2R + MN, Pla2r-asscociated idiopathic MN, n = 25; IgAN, n = 23. p, 0.001; NS, not significant

IgAN was characterized by mesangial Gd-IgA1 deposition and elevated serum Gd-IgA1 levels. As illustrated in Figure 2(A), in kidney biopsies of cIgA-MN patients, Gd-IgA1 deposition was absent in the glomerulus. In the context of MN, patients exhibiting mesangial IgA co-deposition demonstrated immune characteristics comparable to those observed in primary IgAN patients. Notably, this similarity was hallmarked by the co-localization of Gd-IgA1 alongside IgA within the mesangium, as evidenced by kidney biopsy findings. This co-localization suggests a shared pathogenic mechanism that warrants further investigation to elucidate the underlying immunological processes in these distinct yet related renal conditions. Finally, Gd-IgA1 levels were comparable between MN patients with or without capillary IgA and IgG co-deposition but were significantly lower than those of IgAN patients (Figure 2(B)).

### Co-deposition of IgA and Pla2r

**Figure 3.**
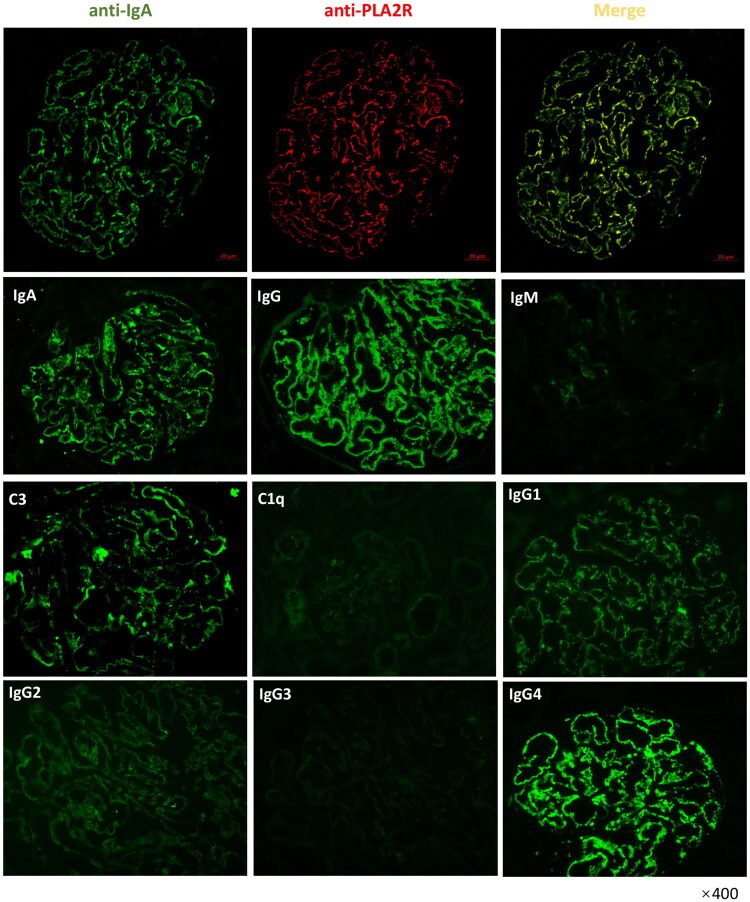
Positive IgA staining along capillary walls in Pla2r-associated membranous nephropathy. Representative images of IgA, Pla2r, IgM, C3, C1q, IgG and IgG1-4 staining of renal biopsy of MN patients. Indirect immunofluorescence of IgA and Pla2r were performed on paraffin-embedded kidney specimen and direct immunofluorescence of IgA, IgG, IgM, C3, C1q, IgG1-4 were performed on frozen sections of fresh kidney tissue.

Pla2r-associated MN was the most common type of primary MN and was characterized by IgG and Pla2r staining along the glomerular capillary loop, as demonstrated by immunofluorescence. As displayed in Figure 3, immunofluorescence analysis of kidney biopsies from Pla2r-positive cIgA-MN patients revealed that IgA completely overlapped with Pla2r along the glomerular capillary loop and deposited in the same granular pattern as Pla2r, IgG, C3, IgG1, and IgG4.

### Characterization of IgA subtype of Pla2r-antibody

**Figure 4.**
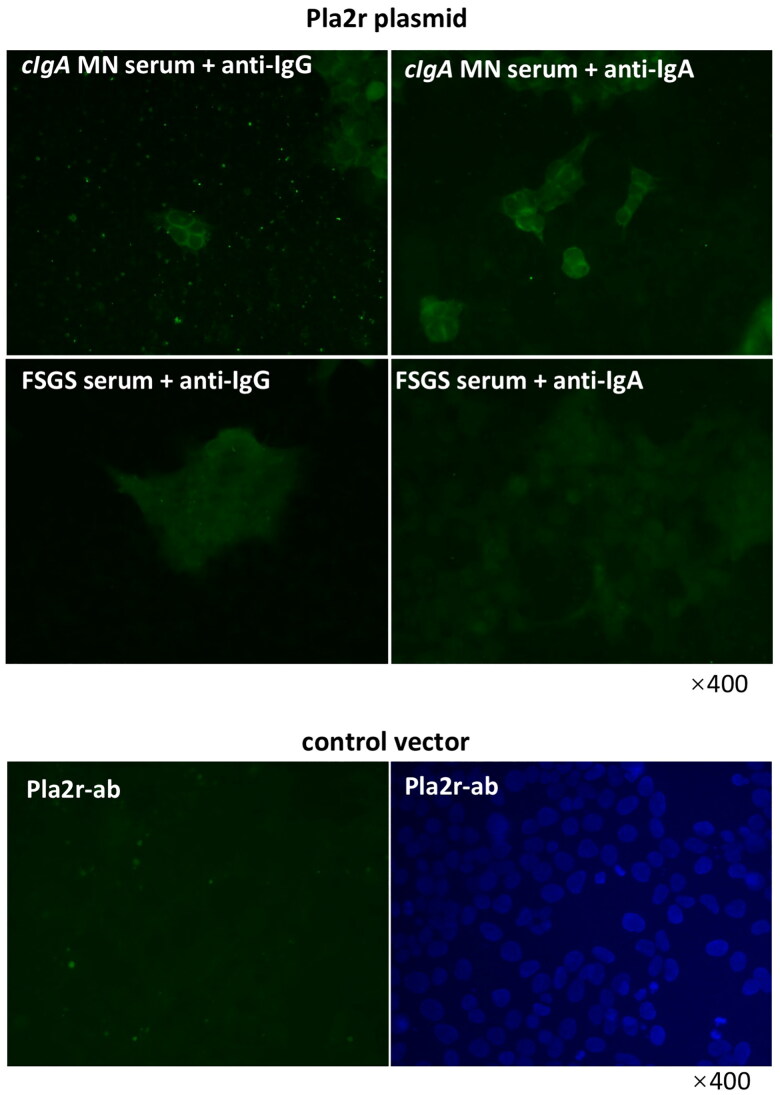
IgA subtype present in serum anti-Pla2r antibody. Serums from patients diagnosed Pla2r-associated iMN with capillary IgA deposition and FSGS were incubated with HEK 293 cells transfected with a PLA2R isoform 1 full length cDNA. Fitc- anti-human IgA and fitc-anti-human IgG were used as secondary antibody respectively. Rabbit anti Pla2r antibody were incubated with HEK 293 cells transfected with a Pla2r cDNA or control vector as control.

Circulating Pla2r-antibodies and their classes were first detected by indirect immunofluorescence analysis using HEK293 cells transfected with Pla2r plasmid, as depicted in Figure 4. As anticipated, both IgA and IgG were positive for Pla2r-antibody in sera from Pla2r-associated cIgA-MN patients. Conversely, sera from focal segmental glomerulosclerosis patients, serving as negative controls, were negative for Pla2r-IgA and IgG. The control vector and commercially available Pla2r antibody served as controls.

**Figure 5.**
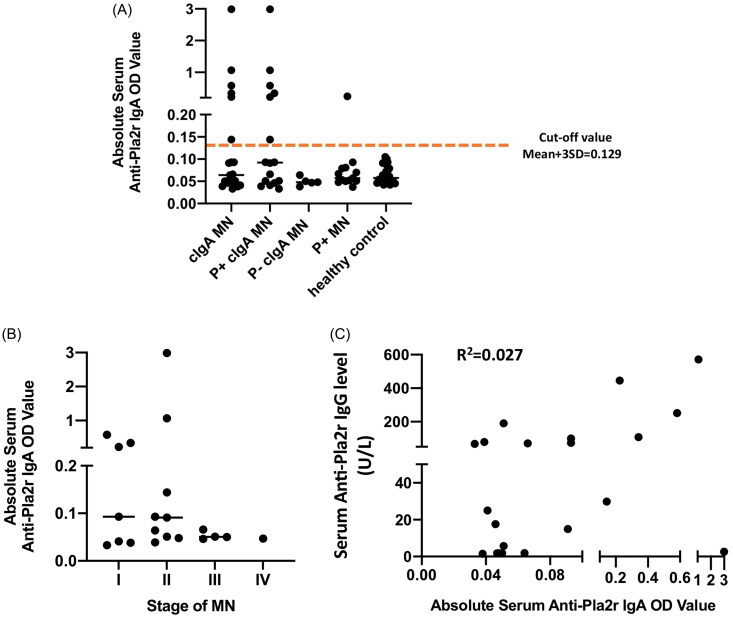
Determination of serum anti-Pla2r IgA by ELISA. Sera from patients were assayed for Pla2r of IgG and IgA subtype. Pla2r-IgA results were documented in absolute O.D. values. (A) Serum Pla2r IgA results from MN and health controls. cIgA MN, patients diagnosed with iMN with capillary IgA deposition n = 21; P + cIgA MN, patients diagnosed with Pla2r-accosicated iMN with capillary IgA deposition n = 16; P- cIgA MN, patients diagnosed with Pla2r-negative iMN with capillary IgA deposition n = 5; P + MN, Pla2r-accosicated iMN, n =13; healthy control, n = 24. (B) Serum Pla2r IgA results from 21 patients diagnosed iMN with capillary IgA deposition classified accroding to stage of MN. (C) Correlation analysis of serum Pla2r IgG and IgA results from 21 patients diagnosed iMN with capillary IgA deposition.

Furthermore, ELISA was performed to further investigate the prevalence and type of Pla2r across different classes of MN, and IgA O.D. values above the cutoff value (mean + 3SD values of the healthy controls) were considered positive. Serum samples were available from 16 Pla2r-associated cIgA-MN patients and 13 Pla2r-associated MN without IgA co-deposition for ELISA. As delineated in Figure 5(A), 37.5% (6/16) of Pla2r-accosted cIgA-MN patients and 7.7% (1/13) of patients with Pla2r-associated MN without IgA co-deposition tested positive for serum Pla2r-IgA antibodies, whereas no Pla2r-negative cIgA-MN patients tested positive. As presented in Figure 5(B) and (C), and Supplementary Figure 1, Pla2r-IgA positivity was more prevalent in patients with Stages I and II MN, and O.D. values of Pla2r-IgA were independent of Pla2r-IgG levels.

**Figure 6.**
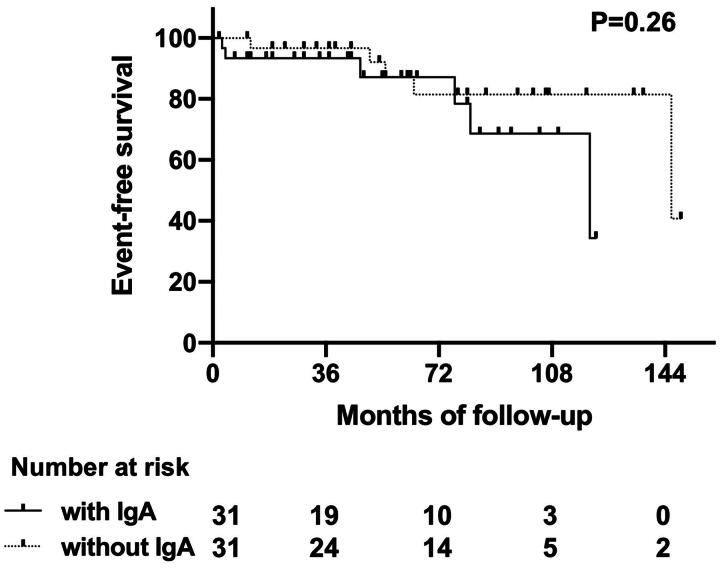
Kaplan-Meier plots for outcomes of iMN patients with or without capillary IgA deposition. Event-free survival for iMN patients with or without capillary IgA deposition was not significant different. Event was defined as eGFR reduction >50% from baseline, ESKD or all-cause mortality.

### Treatment response and outcome

Among the 34 cIgA-MN patients, 31 (91.2%) had completed follow-up data, as listed in Table 3. An additional 31 patients from the idiopathic MN cohort without capillary IgA deposition were matched using PSM and served as controls. The mean follow-up duration of the control group was 63 months (IQR 37-102), which was longer than that of the observation group (44 months, IQR 17-82). A total of 20 patients from the MN cohort without capillary IgA deposition were administered one form of immunosuppressive (IS) therapy (including 10 patients who received over one type of IS agent), while 13 patients in the cIgA-MN group were treated with IS agents and 3 patients with more than one type of IS agents, as detailed in Supplementary Table 2. However, no significant difference was noted in the proportion of patients receiving immunosuppressive therapy between the two groups (41.9% vs 64.5%). The remission rate was 64.5% in the observation group and 80.6% in the control group. One patient died of cerebral hemorrhage, and two patients progressed to End-stage kidney disease (ESKD) in the observation group, whereas only one patient progressed to ESKD in the control group. eGFR reduction > 50% from baseline was identified in three patients in the observation group and fore in the control group. Further analysis using Kaplan-Meier plots, as shown in Figure 6, unveiled no significant differences in outcomes between iMN patients with or without capillary IgA deposition.

**Table 3.** Treatment and follow-up of iMN patients with or without capillary IgA deposition.

Treatment and follow-up	iMN with capillary IgA deposition *N* = 31	iMN without capillary IgA deposition* *N* = 31	*p* value	OR (95% CI)
Age (years)	50 (30, 66)	54 (49, 60)	0.54	
Men, n(%)	21 (67.8)	23 (75.2)	0.58	
Initial proteinuria (g/day)	6.0 (2.3, 9.8)	6.8 (3.8, 12.4)	0.43	
eGFR (mL/min/1.73 m^2^)	99 (85, 119)	93 (72, 102)	0.11	
Immunosuppressive therapy	13 (41.9)	20 (64.5)	0.13	
Follow-up interval (months)^a^	44 (17,82)	63 (37, 102)	0.06	
Complete remission, n(%) ^b^	14 (45.2)	14 (45.2)	>0.99	1.0 (0.35 to 2.88)
Partial remission, n(%) ^b^	6 (19.4)	11 (35.5)	0.25	0.44 (0.13 to 1.38)
No remission, n(%)	11 (35.5)	6 (19.4)	0.25	2.29 (0.72 to 7.86)
ESKD or all-cause mortality, n(%)	3 (9.8)	1 (3.2)	0.61	3.21 (0.45 to 42.94)
eGFR reduction > 50% from baseline, n (%)	3 (9.8)	4 (12.9)	>0.99	0.72 (0.17 to 2.92)

^*^ Matched by PSM from 180 iMN patients without capillary IgA deposition.

Age, gender, initial proteinuria, eGFR were set as predictor and match tolerance/caliper was 0.03.

Data are presented as median (interquartile-range) or number of cases (% of total cases).

OR, odds ratio; CI, confidence intervals.

^a^ Follow-up data were available in 31 of the 34 iMN patients with capillary IgA deposition.

^b^ Partial remission was defined as proteinuria <3.5 g/day plus a 50% reduction from its peak value; complete remission was defined as proteinuria <0.3 g/day.

## Discussion

This study aimed to describe the characteristics of cIgA-MN and identify long-term prognostic factors. Among the 531 cases of MN diagnosed in our center from January 2007 to May 2020, 53 patients exhibited moderate IgA deposition on the capillary walls along with IgG. Electron microscopy uncovered the presence of electron-dense deposits located in the subepithelial region rather than the mesangial region. Nineteen patients were excluded from the study due to underlying medical conditions such as lupus, Sjogren’s syndrome, psoriasis, hepatitis B infection, kidney transplantation, amyloid nephropathy, and chemical exposure. Of note, careful consideration and exclusion of patients with these medical conditions are essential to ensure the validity and reliability of the research findings.

Since 1983, a substantial body of research has documented the simultaneous presence of IgA in the mesangial area and IgG deposition on the glomerular capillary wall in certain patients [10–12]. This phenomenon has been hypothesized to be indicative of superimposed membranous nephropathy (MN) on an underlying mild IgA nephropathy. Herein, renal immunostaining for Gd-IgA1 was performed using the KM55 antibody, and the plasma levels of Gd-IgA1 were determined using the KM55 ELISA kit (Immuno-Biological Laboratories). As anticipated, our cohort exhibited negative KM55 staining in renal tissues and very low levels of plasma Gd-IgA1, similar to those observed in MN individuals without IgA deposits. These results collectively suggest that the presence of capillary IgA deposits with IgG in MN does not contribute to Gd-IgA1 deposition. There are several variants of glomerular disease with IgA deposits combined with capillary wall abnormalities. IgA-dominant infection-related glome­rulonephritis may present with prominent subepithelial dense deposits, although these deposits typically display a hump-like appearance [3]. However, there was no evidence of concurrent infection or a pattern of injury suggestive of IgA-dominant infection-related glomerulonephritis in this cohort.

As is well documented, Pla2r is an autoantigen present in glomerular podocytes. In 2009, Beck et al. discovered that the extracellular domain of Pla2r acts as a mutant antigen that activates autoimmune responses [13]. In conjunction with anti-Pla2r IgG antibodies, especially IgG4, an *in situ* immune complex is formed, which activates the complement system and elicits podocyte injury, a key pathogenic mechanism for the majority of primary MN patients. Serum Pla2r antibody is highly specific for the diagnosis of primary MN and can distinguish primary MN from other glomerular diseases [14]. IgG is the most common class of antibodies in blood and tissues and plays a crucial role in the immune response to pathogens and foreign invaders. IgG antibodies have a greater ability to bind to antigens and trigger immune responses, rendering them more efficient at targeting and neutralizing self-antigens in autoimmune diseases [15]. However, the presence of IgA-type anti-Pla2r antibodies in a subset of cIgA-MN patients is a novel finding, signaling a potential role for IgA in the pathogenesis of Pla2r-associated MN, which has not been extensively explored in earlier studies. IgA deposits overlapped spatially with Pla2r and IgG4 in subepithelial immune complexes, indicating shared antigen targeting. Besides, IgA anti-Pla2r antibodies were more frequently observed in early-stage MN (Stage I/II), implying a role in initial immune recognition. Pla2r is a transmembrane glycoprotein with a large extracellular domain rich in conformational epitopes. The predominance of IgG4 antibodies in MN can be ascribed to their unique ability to form small, non-complement-fixing immune complexes. The Fab region of IgA, which shares high homology with IgG in antigen-binding domains, may recognize pla2r epitopes, as demonstrated by the IIF and ELISA results. Nonetheless, it is worthwhile acknowledging that not all antibodies associated with immune diseases are of the IgG type; IgA antibodies have also been reported. For instance, studies investigating ANCA vasculitis established that IgA anti-PR3 is present in approximately 30% of patients, especially those with upper airway involvement [16]. Additionally, IgA anti-MPO can be detected in certain patients and may be more commonly detected during periods of active disease [17]. IgA anti-dsDNA antibodies correlate with renal flares, highlighting the pathogenic potential of systemic IgA [18]. Taken together, these examples validate that IgA autoantibodies can target systemic antigens independent of mucosal triggers.

Herein, fluorescence microscopy images of patients with Pla2r -associated cIgA-MN revealed granular positivity in the immunofluorescence labeling of both Pla2r-IgG and IgA along the glomerular basement membrane. Subsequently, serum samples from these patients and healthy controls were incubated with HEK 293 cells transfected with Pla2r cDNA, and the results indicated the presence of not only IgG antibodies but also IgA antibodies that were positive for Pla2r. Furthermore, the proportion of circulating IgA-type anti-Pla2r antibodies in patients with cIgA-MN, as detected by ELISA, was generally higher than that in patients with primary MN without capillary IgA deposition, although the difference did not reach statistical significance due to the limited number of patients. More importantly, these results indicate a potential link between IgA and Pla2r in the pathogenesis of membranous nephropathy. Meanwhile, no correlation was identified between the serum levels of IgA or IgG-type anti-Pla2r antibodies in patients with cIgA-MN. There have been three reported cases of Pla2r-negative membranous nephropathy with single polyclonal granular IgA deposition along the glomerular capillary walls without IgG involvement [19–21]. Specifically, three Asian patients presented with nephrotic syndrome while maintaining preserved renal function. Notably, anti-nuclear antibodies, cryoglobulin, and monoclonal proteins were not detected in any of these cases. These patients exhibited consistent membranous features characterized by solitary IgA immune deposits. The presence of both κ- and λ-light chains along the glomerular capillary walls further substantiated the polyclonal nature of these IgA immune deposits. However, among the three cases of MN displaying solely capillary IgA deposition, Pla2r renal fluorescent staining was not reported in one case and yielded negative results in the other two. Consequently, we postulate that exposure to alternative antigens on podocytes may induce the production of IgA autoantibodies, differentiating these cases from the findings in our patient cohort. Therefore, the clinical presentation of our patients cannot be categorized alongside these three reported cases.

The evolving understanding of target antigens in MN presents both opportunities and challenges for contemporary studies. While our investigation focused on the novel immunophenotype of Pla2r-associated IgG/IgA co-deposition, the absence of comprehensive testing for alternative antigens, including Thrombospondin type 1 containing 7A domain (THSD7A), Neural epidermal growth factor-like 1 protein (NELL-1), and other recently identified targets, warrants explicit consideration. Although THSD7A accounts for 3-5% of PLA2R-negative MN cases, its detection relies on specialized immunohistochemistry, and ELISA assays are unavailable in most clinical laboratories [22]. For newer antigens such as NELL-1, Exostosin1/Exostosin2 (EXT1/EXT2), and Semaphorin3B (Sema3B), diagnostic validation remains incomplete [5]. While mass spectrometry identified these proteins in glomerular immune deposits, standardized methods for detecting circulating antibodies or validating their pathogenic role in clinical cohorts are still emerging. The features of our cohort were in line with classical Pla2r-associated MN, contrasting with reported NELL-1-positive cases that frequently exhibit female predominance, malignancy associations, or distinctive ultrastructural patterns [23]. The absence of FDA-cleared or CE-marked commercial assays for these targets, combined with their low individual prevalence (<3% of primary MN), rendered systematic evaluation impractical in our resource-constrained setting. While novel antigens such as Neural cell adhesion molecule 1 (NCAM1), High-temperature requirement A serine peptidase 1 (HTRA1), and Protocadherin 7 (PCDH7) expand the molecular taxonomy of MN, their clinical translation remains hindered by unresolved questions [5].

Besides the circulation, IgA antibodies are present in mucosal tissues, including the nose, respiratory tract, mouth, gastrointestinal system, eyes, ears, and vagina, where they play a key role in protecting against pathogens entering the body through these routes [15]. Herein, only 4 of cIgA-MN patients had a documented history of upper respiratory tract disease prior to disease onset. Evidently, the presence of IgA antibodies against Pla2r in these MN patients may be less related to mucosal immune activation. Further research is needed to explore the implications of IgA deposition in the pathogenesis of MN.

In our investigation, further investigation and comparison of histological changes across various stages of MN revealed that the levels of serum IgA-type anti-Pla2r antibodies were elevated in patients at stages I and II, which suggests that IgA antibodies may be present during the early phases of Pla2r-associated MN. Previous studies have pointed out that in certain autoimmune diseases, such as lupus, IgA autoantibodies are detectable during the early stages of the disease. Research has indicated that pediatric SLE patients with active disease exhibit significantly higher levels of circulating IgA autoantibodies compared to healthy individuals, while those with clinically inactive disease have intermediate levels [24]. As is well established, IgA is less stable than IgG and is thus present in lower levels in the serum at any given time. Additionally, IgG antibodies have a longer half-life in the body compared to IgA antibodies, allowing them to persist for longer periods of time and continue to target self-antigens [15]. The precise role of systemic IgA remains to be elucidated. Research has shown that the binding of monomeric IgA in serum to FcαRI triggers immune complexes that activate neutrophils, monocytes, and CD103^+^ dendritic cells expressing FcαRI, leading to inflammatory responses [25,26]. The presence of IgA deposits in membranous nephropathy glomeruli may indicate a more complex immune response in the pathogenesis of this condition. Nevertheless, the exact role of systemic IgA in autoimmune diseases remains unknown.

No significant difference in prognosis was observed between patients with cIgA-MN and those with Pla2r-positive MN without IgA deposition. As shown in Table 3 and Supplementary Table 2, cIgA-MN patients received a higher proportion of non-immunotherapy supportive treatment, and when immunotherapy was administered, it primarily consisted of single-agent regimens. Despite these differences in treatment, the prognosis of the cIgA-MN and control groups were similar, indicating that cIgA-MN patients presented with early-stage disease and less severe conditions. Additionally, no abnormalities were detected in terms of clinical remission. It is widely recognized that MN generally has a favorable prognosis, with a low end-stage kidney disease (ESKD) rate of only 3% over a 10-year follow-up period. Nevertheless, several limitations of this study merit acknowledgment. To begin, its single-center design may limit the generalizability of the findings. Secondly, the relatively small cohort size of 34 primary cIgA-MN cases may not provide sufficient statistical power to detect subtle differences in clinical outcomes or treatment responses. Therefore, future multicenter studies with larger cohorts are required to validate our findings and further explore the implications of IgA deposition in the pathogenesis of MN. In addition, the follow-up duration of 63 months (IQR 37-102) may have been insufficient to observe significant differences in long-term outcomes, given the generally favorable prognosis of MN. Extending the follow-up period in future studies could assist in elucidating the true impact of IgA deposition on the prognosis of Pla2r-associated MN.

## Supplementary Material

SFigure1.jpeg

## References

[1] Suzuki H, Yasutake J, Makita Y, et al. IgA nephropathy and IgA vasculitis with nephritis have a shared feature involving galactose-deficient IgA1-oriented pathogenesis. Kidney Int. 2018;93(3):700–705. doi: 10.1016/j.kint.2017.10.019. 29329643

[2] Bridoux F, Javaugue V, Nasr SH, et al. Proliferative glomerulonephritis with monoclonal immunoglobulin deposits: a nephrologist perspective. Nephrol Dial Transplant. 2021;36(2):208–215. doi: 10.1093/ndt/gfz176. 33494099

[3] Paueksakon P, Najafian B, Alpers CE, et al. AJKD atlas of renal pathology: IgA-Dominant infection-related glomerulonephritis. Am J Kidney Dis. 2024;83(1):e1–e2. doi: 10.1053/j.ajkd.2023.05.009. 38129071

[4] Couser WG. Primary membranous nephropathy. Clin J Am Soc Nephrol. 2017;12(6):983–997. doi: 10.2215/CJN.11761116. 28550082 PMC5460716

[5] Sethi S, Fervenza FC. Membranous nephropathy-diagnosis and identification of target antigens. Nephrol Dial Transplant. 2024;39(4):600–606. doi: 10.1093/ndt/gfad227. 37863839

[6] Qin W, Beck LH, Jr., Zeng C, et al. Anti-phospholipase A2 receptor antibody in membranous nephropathy. J Am Soc Nephrol. 2011;22(6):1137–1143. doi: 10.1681/ASN.2010090967. 21566055 PMC3103733

[7] Inker LA, Eneanya ND, Coresh J, et al. New creatinine- and cystatin C-based equations to estimate GFR without race. N Engl J Med. 2021;385(19):1737–1749. doi: 10.1056/NEJMoa2102953. 34554658 PMC8822996

[8] Cattran DC, Kim ED, Reich H, et al. Toronto glomerulonephritis registry G: membranous nephropathy: quantifying remission duration on outcome. J Am Soc Nephrol. 2017;28(3):995–1003. doi: 10.1681/ASN.2015111262. 27756808 PMC5328151

[9] Xie Q, Li Y, Xue J, et al. Renal phospholipase A2 receptor in hepatitis B virus-associated membranous nephropathy. Am J Nephrol. 2015;41(4-5):345–353. doi: 10.1159/000431331. 26087695

[10] He J-W, Cui D-F, Zhou X-J, et al. Concurrent IgA nephropathy and membranous nephropathy, is it an overlap syndrome? Front Immunol. 2022;13:846323. doi: 10.3389/fimmu.2022.846323. 35359934 PMC8961684

[11] Jennette JC, Newman WJ, Diaz-Buxo JA. Overlapping IgA and membranous nephropathy. Am J Clin Pathol. 1987;88(1):74–78. doi: 10.1093/ajcp/88.1.74. 3300266

[12] Stokes MB, Alpers CE. Combined membranous nephropathy and IgA nephropathy. Am J Kidney Dis. 1998;32(4):649–656. doi: 10.1016/s0272-6386(98)70031-9. 9774129

[13] Beck LH, Jr., Bonegio RG, Lambeau G, et al. M-type phospholipase A2 receptor as target antigen in idiopathic membranous nephropathy. N Engl J Med. 2009;361(1):11–21. doi: 10.1056/NEJMoa0810457. 19571279 PMC2762083

[14] Kidney Disease. Improving Global Outcomes Glomerular Diseases Work G: KDIGO 2021 clinical practice guideline for the management of glomerular diseases. Kidney Int. 2021;100(4S):S1–S276. 34556256 10.1016/j.kint.2021.05.021

[15] Justiz Vaillant AA, Jamal Z, Patel P, et al. Immunoglobulin. Treasure Island (FL): StatPearls; 2024.

[16] Kelley JM, Monach PA, Ji C, et al. IgA and IgG antineutrophil cytoplasmic antibody engagement of Fc receptor genetic variants influences granulomatosis with polyangiitis. Proc Natl Acad Sci U S A. 2011;108(51):20736–20741. doi: 10.1073/pnas.1109227109. 22147912 PMC3251158

[17] Oommen E, Hummel A, Allmannsberger L, et al. IgA antibodies to myeloperoxidase in patients with eosinophilic granulomatosis with polyangiitis (Churg-Strauss). Clin Exp Rheumatol. 2017;35 Suppl 103(1):98–101. PMC5514423 28281453

[18] Lee AYS. IgA anti-dsDNA antibodies: a neglected serological parameter in systemic lupus erythematosus. Lupus. 2022;31(2):137–142. doi: 10.1177/09612033221074184. 35049409

[19] Li B, Huang H, Yang S, et al. Idiopathic membranous nephropathy with solitary immunoglobulin A deposition: a case report and a review of the literature. Intern Med. 2022;61(13):2019–2025. doi: 10.2169/internalmedicine.8404-21. 34866101 PMC9334231

[20] Sawamura M, Komatsuda A, Kaga H, et al. Membranous nephropathy with solitary polyclonal IgA deposition: A case report and literature review. Clin Nephrol Case Stud. 2019;7(01):60–65. doi: 10.5414/CNCS109807. 31673485 PMC6822057

[21] Kobayashi M, Usui J, Sakai K, et al. Membranous nephropathy with solitary immunoglobulin A deposition. Intern Med. 2015;54(9):1081–1084. doi: 10.2169/internalmedicine.54.3655. 25948352

[22] Tomas NM, Beck LH, Meyer-Schwesinger C, et al. Thrombospondin type-1 domain-containing 7A in idiopathic membranous nephropathy. N Engl J Med. 2014;371(24):2277–2287. doi: 10.1056/NEJMoa1409354. 25394321 PMC4278759

[23] Caza TN, Hassen SI, Dvanajscak Z, et al. NELL1 is a target antigen in malignancy-associated membranous nephropathy. Kidney Int. 2021;99(4):967–976. doi: 10.1016/j.kint.2020.07.039. 32828756 PMC7895854

[24] Ukadike KC, Ni K, Wang X, et al. IgG and IgA autoantibodies against L1 ORF1p expressed in granulocytes correlate with granulocyte consumption and disease activity in pediatric systemic lupus erythematosus. Arthritis Res Ther. 2021;23(1):153. doi: 10.1186/s13075-021-02538-3. 34051843 PMC8164314

[25] Hansen IS, Krabbendam L, Bernink JH, et al. FcalphaRI co-stimulation converts human intestinal CD103(+) dendritic cells into pro-inflammatory cells through glycolytic reprogramming. Nat Commun. 2018;9(1):863. doi: 10.1038/s41467-018-03318-5. 29491406 PMC5830413

[26] Hansen IS, Hoepel W, Zaat SAJ, et al. Serum IgA immune complexes promote proinflammatory cytokine production by human macrophages, monocytes, and Kupffer cells through FcalphaRI-TLR cross-talk. J Immunol. 2017;199(12):4124–4131. doi: 10.4049/jimmunol.1700883. 29118246

