# Supplementary figures and images for: Characteristics of adult membranous nephropathy patients with capillary co-deposition of IgG and IgA
Source: Ren Fail. 2025 Jun 24;47(1):2512393. doi: 10.1080/0886022X.2025.2512393 (PMC12893482; doi:10.1080/0886022X.2025.2512393)

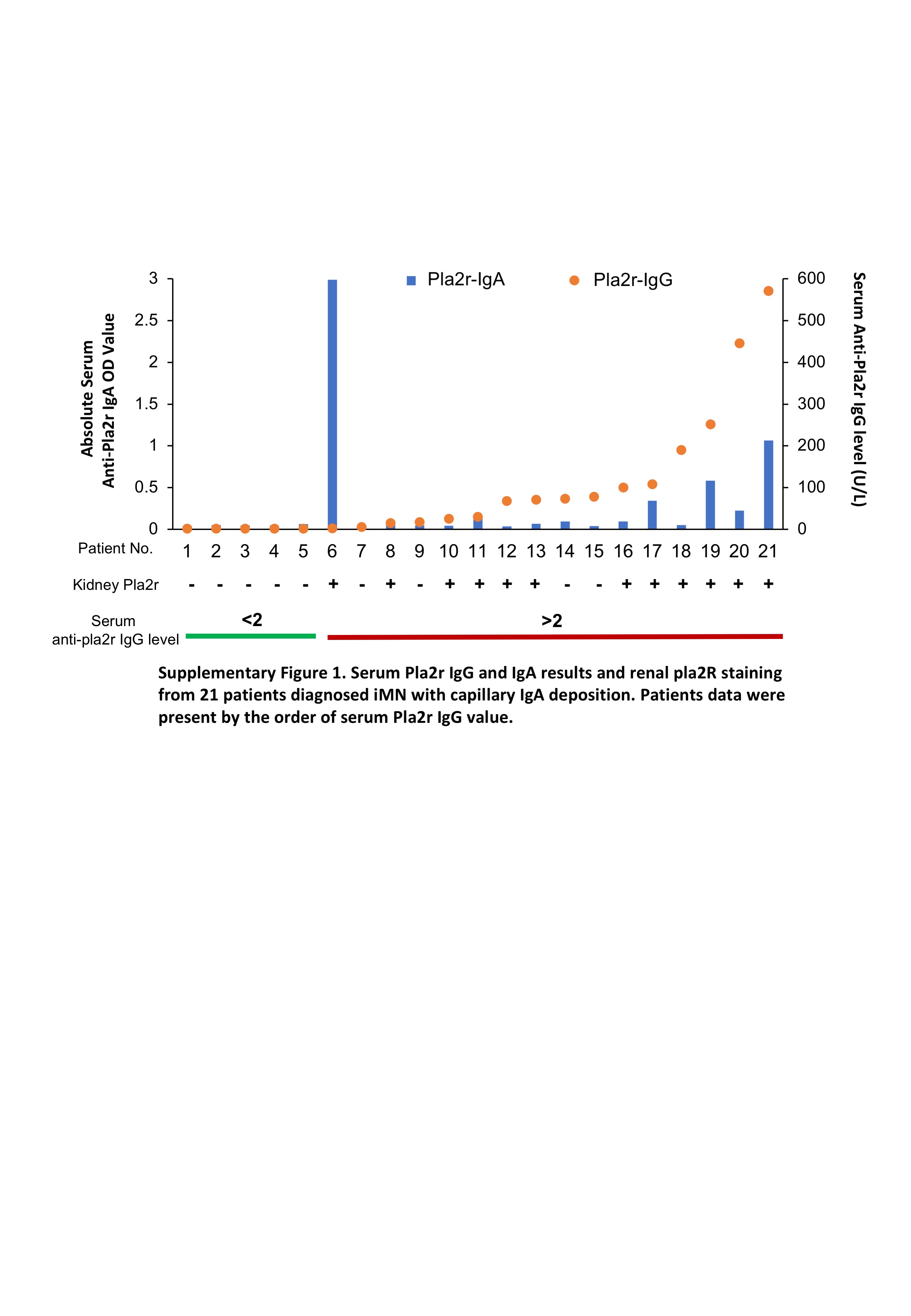

Supplement: SFigure1.jpeg [file IRNF_A_2512393_SM6145.jpeg]
